# Pathogen-Driven Selection in the Human Genome

**DOI:** 10.1155/2013/204240

**Published:** 2013-03-04

**Authors:** Rachele Cagliani, Manuela Sironi

**Affiliations:** Bioinformatics, Scientific Institute IRCCS E. Medea, 23842 Bosisio Parini (LC), Italy

## Abstract

Infectious diseases and epidemics have always accompanied and characterized human history, representing one of the main causes of death. Even today, despite progress in sanitation and medical research, infections are estimated to account for about 15% of deaths. The hypothesis whereby infectious diseases have been acting as a powerful selective pressure was formulated long ago, but it was not until the availability of large-scale genetic data and the development of novel methods to study molecular evolution that we could assess how pervasively infectious agents have shaped human genetic diversity. Indeed, recent evidences indicated that among the diverse environmental factors that acted as selective pressures during the evolution of our species, pathogen load had the strongest influence. Beside the textbook example of the major histocompatibility complex, selection signatures left by pathogen-exerted pressure can be identified at several human loci, including genes not directly involved in immune response. In the future, high-throughput technologies and the availability of genetic data from different populations are likely to provide novel insights into the evolutionary relationships between the human host and its pathogens. Hopefully, this will help identify the genetic determinants modulating the susceptibility to infectious diseases and will translate into new treatment strategies.

## 1. Infections: A Scourge throughout Human History

Infectious diseases and epidemics have always accompanied and characterized human history, representing one of the main causes of death. Even today, despite progress in sanitation and medical research, infectious diseases represented a major killer; data published by the World Health Organization (WHO 2008, http://www.who.int/en/) indicate that about 15% of deaths in the world's population are due to infectious and parasitic diseases, reaching about 41% in Africa. In the most recently published report (November 2012), the WHO estimated that in 2011 about 4.4 millions of children younger than 5 years died of infection. 

Anatomically modern humans appeared in East Africa about 200,000 years ago, spread out from sub-Saharan Africa approximately 100,000 years ago, and subsequently colonized the rest of the world in a series of migratory events [1]. During this process, humans not only encountered a wide range of different environmental conditions, including diverse pathogen species, but also introduced changes in subsistence strategies that allowed the development of large, interconnected societies. Hunter/gatherer communities are thought to have suffered from infections with specific characteristics, that favor the maintenance of the agent in a small population. Wolfe and colleagues [2] identify these characteristics as (i) the presence of animal reservoirs; (ii) incomplete immunity, enabling previously infected subjects to remain in the pool of potential victims; (iii) a slow or chronic disease course, so that infected members can infect new victims over years. Thus, early humans possibly hosted a limited array of pathogenic species. However, the development of agriculture about 11,000 years ago led to a set of changes that favored the establishment of the most important epidemic diseases [2]. In particular, the domestication of animals and a sedentary lifestyle led to a steady increase in the exposure to zoonotic infections [3, 4]. Indeed, many of the most important pathogens in human history, such as smallpox and human morbillivirus (measles), evolved from diseases of domestic animals [3]. The presence of domestic herds is also thought to have increased human contact with vectors carrying malaria, yellow fever, dengue, trypanosomiasis, and filariasis. 

With the development of urban centers, in about 3,000 BC, and increased communication between neighboring towns, human settlements became large enough to maintain diseases in an endemic form. This is the case, for example, for measles; for the longest part of its history, measles has been preserved by sequential outbreaks in rural villages, until the density of human populations was large enough to facilitate and support the presence of the infectious agent as an endemic pathogen [3]. The development of urban centers has also been accompanied by human expansion into new areas. The migration of Europeans in the New World during the fifteenth and sixteenth centuries is an example of the effect of population movement on pathogen transmission [5]. On the one hand, novel diseases, such as smallpox, measles, and typhus, were introduced in South and Central America, and the indigenous population, who had no or little natural resistance or immunity to these agents, were decimated: the population of Mexico was reduced from 20 millions to 3 millions between 1518 and 1568. On the other, shortly after Columbus's return from the New World, syphilis epidemics began to be recorded across Europe: although the issue is still debated, *Treponema pallidum* is thought to have been introduced to the Old World by crews returning from the Americas [6].

Human history has a rich record of epidemic and pandemic examples: from the plague of biblical times, through smallpox epidemics in Rome in the second and third centuries AD, to flu and bubonic plague epidemics. In relatively recent historical times, the 1918–1920 Spanish flu pandemic caused by far the highest mortality recorded in history: medical historians have estimated at least 20 million deaths [5]. 

Advances in medical science, the use of antibiotics, and vaccines, as well as improved hygienic conditions, have contributed to reduce the burden of infectious diseases and to eradicate some deadly pathogens (e.g., smallpox). Nonetheless, we have been witnessing the appearance and rapid spread of novel epidemics, first and foremost HIV/AIDS. 

## 2. A Wide Spectrum of Selection Targets

Haldane, a pioneer in many fields of science, and in genetics in particular, was among the first to propose that, as infectious diseases had been a major threat to human populations, they had been acting as a powerful selective pressure and may be considered a major driver of evolution in our species [7]. A few years after this initial observation, he suggested that the high prevalence of thalassemia in the Mediterranean basin was the result of a selective pressure imposed by malaria [8]. We now know Haldane's hypotheses to be true, but it was not until the availability of large-scale genetic data and the development of novel methods to study molecular evolution that we could assess how strongly and pervasively infectious agents have shaped our genome(s). In fact, a recent genome-wide analysis has indicated that among the diverse environmental factors that most likely acted as selective pressures during the evolution of our species (climate, diet regimes, and infections), pathogen load had the strongest influence on the shaping of human genetic variability [9]. In their work, Fumagalli and coworkers [9] also indicated that the effect of distinct pathogens can at least in part be disentangled, although infectious agents were grouped into macrocategories (i.e., viruses, bacteria, helminths, and protozoa). Indeed, in his observations about thalassemia, Haldane chose a lucky rare example: the specific characteristics of *Plasmodium* infection and the strong selective pressure the parasite exerted allowed to draw a direct link between the infectious agent and specific loci showing very strong evidence of natural selection (e.g., *DARC* and *G6PD* [10, 11]). This has proved unfeasible for the large majority of human genes, whereby signatures of natural selection are described, but the underlying selective pressure (i.e., the specific pathogen) remains unknown. The case of thalassemia proposed by Haldane also points to another important aspect of pathogen-driven evolution: genes directly involved in immune response do not represent the only selection targets. Indeed, thalassemia is caused by inherited mutations of globin genes, which do not encode immune response effectors; nonetheless, HbS, the *HBB* allele responsible for sickle cell hemoglobin, is maintained by balancing selection at a frequency of about 10% in regions where *Plasmodium* is endemic because heterozygotes have a greatly reduced risk of developing severe malaria (reviewed in [12]). No direct involvement in immune response is also observed, for example, for glycophorins A and C (GYPC and GYPA), sialoglycoproteins abundantly expressed on the surface of red blood cells and exploited by *Plasmodium falciparum* for erythrocyte invasion [13, 14]. Both *GYPC* and *GYPA* have evolved adaptively in human populations, the underlying pressure being most likely accounted for by malaria [15–17]. Genetic variability at the *GYPA* and *GYPC* loci is responsible for the MNS and Gerbich blood group systems, respectively (Blood Group Antigen Gene Mutation Database (BGMUT) [18]). In humans, as many as 29 blood group systems have been described to date, and the molecular basis for most of them has been identified (Blood Group Antigen Gene Mutation Database (BGMUT) [18]). In the majority of cases, the genes specifying these systems encode a glycosyltransfrease or a glycosylated protein with an expression that is not limited to the erythrocyte. Here, again, Haldane had his say when he suggested that antigens constituted of glycoproteins account for a “surprising biochemical diversity by serological tests” and possibly play a role in resistance/predisposition to pathogen infection [19]. The *ABO* gene encodes a glycosyltransferase, and the ABO histoblood group was one of the first human polymorphisms identified. At the beginning of the twentieth century, the wide variability of A and B blood group frequencies across human populations had already been noticed [20]. Since then, we have learned that *ABO* polymorphisms have been maintained in human populations by the action of balancing selection, and that the null allele responsible for the O group has appeared at least three times during the evolution of our species [21, 22]. Indeed, a recent work indicated that *ABO* polymorphisms are transspecific (i.e., they are shared by descent among different primate species) and have been preserved by selection for millions of years, a very rare phenomenon outside the MHC (see the following) [23]. The expression of ABO histo-blood group antigens on the gastrointestinal mucosa and in bodily secretions (secretor phenotype) depends on the action of a fucosyltransferase encoded by *FUT2* (Lewis blood group system); in different human populations distinct *FUT2* polymorphisms make a portion of subjects “nonsecretors;” these variants have undergone distinct selective patterns in different geographic areas, ranging from long-standing balancing selection to recent selective sweeps, and suggest convergent evolution for the nonsecretor phenotype [24]. The evolutionary history of both *ABO* and *FUT2* points to a long-lasting and widespread selective pressure: this is at least partially accounted for by infectious agents. Indeed, the susceptibility to a number of pathogens, including *Plasmodium falciparum* [25, 26], Norwalk virus [27], *Campylobacter jejuni* [28], *Helicobacter pylori* [29], and *Vibrio cholerae* [30], is modulated by ABO histoblood group and/or secretor status. In most cases, the different susceptibility to disease or to severe symptoms of disease is explained by the fact that ABO antigens are exploited as attachment sites by specific pathogen-encoded molecules, which, in turn, are subject to a selective pressure for increased ability to infect their host, as recently shown for the *H. pylori babA* gene, encoding the adhesin responsible for ABO antigen binding [31]. Indeed, their functioning as incidental pathogen receptors is thought to be the reason why other genes responsible for blood-histogroup phenotypes have been targeted by pathogen-driven selection in humans [9]. Aside from blood-group antigens, the exploitation of glycoconjugates, with specific preference for sialylated and fucosylated oligosaccharides, by pathogens seems to be common [32]. In line with this view, it has recently been shown that genes involved in the biosynthesis of glycan structures are preferential targets of virus-driven selective pressure [33]. In this respect, an interesting example is accounted for by the *LARGE* gene, encoding a glycosyltransferase that participates in the posttranslational modification of *α*-dystroglycan, a ubiquitous receptor for extracellular matrix proteins. Glycosylation by *LARGE* is critical for binding of arenaviruses of different phylogenetic origin, including Lassa fever virus (LFV) and lymphocytic choriomeningitis virus, to *α*-dystroglycan [34, 35]. LSV causes a deadly hemorrhagic fever and is endemic in West Africa. In a genome-wide screen for recent selective sweeps in humans, a major signal was detected at the *LARGE* locus in populations of African ancestry [36], and the same gene was described to be subject to virus-driven selection in another study [33]. Subsequent analyses indicated that the selection signal is confined to the two first introns of *LARGE*, suggesting that the selection target might be accounted for by one or more polymorphisms that regulate gene expression and consequently *α*-dystroglycan glycosylation and virus binding [37]. This interesting possibility remains to be experimentally verified. 

## 3. Pathogen-Driven Selection at Immune Response Loci

Possibly, the best known example of natural selection in the human genome is accounted for by the major histocompatibility complex (MHC), located on the short arm of chromosome 6 in humans. As in most vertebrates, the human MHC comprises both class I and class II loci that encode molecules directly involved in the presentation of antigens to effector immune cells. The MHC represents the most polymorphic gene cluster in humans, and more than 2,700 alleles have been described for the most variable gene, HLAB (IMGT/HLA Database, European Bioinformatics Institute). KIR (killer-cell immunoglobulin-like receptors) genes, located in a cluster on chromosome 19q13.4, encode interactors of MHC class I molecules and are also highly polymorphic, with individual genes showing allelic variants and distinct haplotypes having a variable *KIR* gene number [38, 39]. 

A clear indication that the extreme variability at classes I and II *HLA* genes is determined by natural selection first came from the analysis of nucleotide substitution patterns; in the antigen-binding region, the rate of amino acid replacement substitutions is higher than that of synonymous substitutions, while the opposite occurs in gene regions not directly involved in antigen recognition [40, 41]. Indeed, several studies have now confirmed that the high level of diversity at MHC genes is the result of both balancing and directional selection [42–46], and *HLA* genes show trans-species polymorphisms and coalescent times that predate the split of the great ape lineages [47, 48]. The role of MHC molecules and their pattern of diversity clearly suggest adaptation to a wide range of pathogen species leading to amino acid diversification of the antigen binding groove. A formal demonstration that pathogens have represented the underlying selective pressure driving HLA class I molecular evolution came from an analysis of geographic diversity of *HLAA*, *HLAB,* and *HLAC* in human populations. Prugnolle and coworkers [49] analyzed 61 populations from different areas of the world and indicated that increased diversity at *HLA* class I genes (compared to the genome average) is observed in populations living in geographic regions where pathogen diversity is also high; the authors indicated that this effect is not merely explained by human demographic history, thus providing an elegant demonstration for a long-standing hypothesis. It is also worth mentioning that pathogen-driven selection on MHC genes has been proposed to influence mate choice in humans, at least in some populations [50]. The underlying idea is that the choice of an MHC-dissimilar mate, possibly through preferences based on body odour, would result in MHC-heterozygous offspring, who would be more resistant to pathogens. This hypothesis is still debated, as both contrasting and supporting evidences have been provided [50, 51]. 

Antigens loaded onto HLA class I molecules are generated from intracellular proteins by the antigen processing pathway, which contributes to shaping the antigenic repertoire displayed at the cell surface. The final step of antigen processing occurs in the endoplasmic reticulum where two aminopeptidases, encoded by *ERAP1* and *ERAP2*, act in concert to trim peptides at their N-terminus [52]. Recent evidences have indicated that both genes have been targets of long-standing balancing selection in humans [53, 54]; as the aminopeptidases contribute to shape the antigenic repertoire displayed by HLA class I molecules, which, in turn, determines the ensuing response, it is tempting to speculate that the underlying selective pressure is represented by infectious agents. In line with this view, human cytomegalovirus (HCMV) encodes a microRNA (miR-US4-1) that specifically targets ERAP1, thus limiting the presentation of HCMV-derived peptides [55], whereas variants in ERAP2 have been associated with altered susceptibility to HIV-1 infection [54]. 

Clearly, natural selection exerted by infectious agents on the immune system is not confined to the MHC and to genes involved in antigen presentation and processing. A very nice example in this respect is represented by *IFITM3* (interferon-induced transmembrane protein 3), a restriction factor for influenza A virus identified in a siRNA screen [56]. The gene has a central importance in protecting humans and mice from influenza, and a human synonymous variant in the gene affects the overall protein availability and the severity of infection [57]. Analysis of selection signatures in the gene indicated that it has been a targeted of recent positive selection in Africa [57]. Interestingly, *IFITM3* seems to restrict other viral infections [56], suggesting that its evolutionary history has been driven by viral pathogens. This example, as well as those relative to the MHC and antigen presentation components, confirms that evolutionary analysis of candidate genes with a known role in immune response may reveal the traces of pathogen-driven selection. Nonetheless, the availability of large-scale data on human genetic diversity (e.g., the HapMap Project, the Human Genome Diversity Cell Line Panel, the 1000 Genomes Project, and others) has opened the possibility to screen the whole human genome for signatures of natural selection. Several analyses (e.g., [58–63]) exploited distinct approaches and invariably came up with the finding that genes involved in immune response are preferential selection targets. Specifically, natural selection was shown to target genes involved in both adaptive and innate immunity, and, following the MHC paradigm, the underlying selective pressure is likely to be accounted for by infectious agents. This hypothesis has been tested by authors that specifically modelled virus- and protozoa-driven selection and detected an overrepresentation of signals at immune response loci [17, 33]. Although valuable in their providing a comprehensive scenario, most of these studies relied on SNP genotype data and, as such, are not ideally suited to identify the specific variants targeted by selection. Resequencing efforts provide better information in this respect, as shown, for example, by an analysis of genetic diversity at NOD-like receptors (NLRs) in humans [64]; these genes encode cytoplasmic microbial sensors and can be divided into two subfamilies, NALP and NAIP, based on domain architecture. By extensive Sanger resequencing and population genetic analyses, Vasseur and coworkers [64] showed that NALP family members have evolved under stronger purifying selection compared to NAIPs, suggesting that the former plays a central nonredundant role, not necessarily limited to immune response. Also, the authors identified specific variants targeted by positive selection; a nice example is represented by the *NLRP1* gene, which has recently been associated with susceptibility to congenital toxoplasmosis [65]: one haplotype carrying 7 nonsynonymous variants has increased in frequency worldwide as a result of positive selection; also, a more recent selective event affected the same gene in populations of European ancestry by targeting the Val1059Met polymorphism [64]. The authors detected natural selection signatures at the promoter region of CIITA, as well; the same variant targeted by selection represents a susceptibility allele for inflammatory diseases including rheumatoid arthritis, multiple sclerosis, and myocardial infarction [66]. Variants in *NLRP1* have also been associated with the susceptibility to autoimmune diseases. Thus, these observations contribute novel evidences to a long-standing hypothesis, whereby, during the evolutionary history of humans, adaptation to infection has been traded off with increased susceptibility to autoimmune and inflammatory conditions. Observations supporting this view have increased in the last few years, and a recent genome-wide analysis of susceptibility to Crohn's disease, a chronic inflammatory disease of the digestive tract, indicated that some susceptibility alleles show signatures of balancing and directional selection, and that a significant overlap exists between Crohn's disease risk variants and those associated with mycobacterial disease (e.g., leprosy) [67]. 

## 4. Concluding Remarks

The ever-increasing availability of human genetic diversity data and the development of methods to study molecular evolution have provided support for a hypothesis formulated long ago, whereby infectious diseases have represented a major threat for human populations and, consequently, have contributed to shaping genetic diversity in our species. The development of high-throughput technologies, the availability of cell line panels from different human populations, and the progress in the fields of clinical immunology and epidemiology will provide novel insights into these issues. Thus, in the coming years we expect to witness further developments in our understanding of the molecular signatures associated with pathogen-driven selection, with special emphasis on the identification of the precise selection targets. Hopefully, these efforts will help us to gain insight into the genetic determinants modulating the susceptibility to infectious diseases that today afflict a large proportion of human subjects, and this knowledge will be translated into novel treatment strategies.

## References

[1] White TD, Asfaw B, DeGusta D (2003). Pleistocene Homo sapiens from Middle Awash, Ethiopia. *Nature*.

[2] Wolfe ND, Dunavan CP, Diamond J (2007). Origins of major human infectious diseases. *Nature*.

[3] Dobson AP, Carper ER (1996). Infectious diseases and human population history. *BioScience*.

[4] Diamond J (1997). *Guns, Germs, and Steel: The Fates of Human Society*.

[5] McNeill WH (1976). *Plagues and People*.

[6] Harper KN, Zuckerman MK, Harper ML, Kingston JD, Armelagos GJ (2011). The origin and antiquity of syphilis revisited: an appraisal of Old World pre-Columbian evidence for treponemal infection. *American Journal of Physical Anthropology*.

[7] Haldane JBS (1932). *The Causes of Evolution*.

[8] Dronamraju K (1990). *Selected Genetic Papers of J.B.S. Haldane*.

[9] Fumagalli M, Sironi M, Pozzoli U, Ferrer-Admettla A, Pattini L, Nielsen R (2011). Signatures of environmental genetic adaptation pinpoint pathogens as the main selective pressure through human evolution. *PLOS Genetics*.

[10] Verrelli BC, McDonald JH, Argyropoulos G (2002). Evidence for balancing selection from nucleotide sequence analyses of human G6PD. *American Journal of Human Genetics*.

[11] Hamblin MT, Thompson EE, Di Rienzo A (2002). Complex signatures of natural selection at the Duffy blood group locus. *American Journal of Human Genetics*.

[12] Kwiatkowski DP (2005). How malaria has affected the human genome and what human genetics can teach us about malaria. *American Journal of Human Genetics*.

[13] Maier AG, Duraisingh MT, Reeder JC (2003). Plasmodium falciparum erythrocyte invasion through glycophorin C and selection for Gerbich negativity in human populations. *Nature Medicine*.

[14] Sim BKL, Chitnis CE, Wasniowska CK, Hadley TJ, Miller LH (1994). Receptor and ligand domains for invasion of erythrocytes by *Plasmodium falciparum*. *Science*.

[15] Wilder JA, Hewett EK, Gansner ME (2009). Molecular evolution of gypc: evidence for recent structural innovation and positive selection in humans. *Molecular Biology and Evolution*.

[16] Baum J, Ward RH, Conway DJ (2002). Natural selection on the erythrocyte surface. *Molecular Biology and Evolution*.

[17] Pozzoli U, Fumagalli M, Cagliani R (2010). The role of protozoa-driven selection in shaping human genetic variability. *Trends in Genetics*.

[18] Blumenfeld OO, Patnaik SK (2004). Allelic genes of blood group antigens: a source of human mutations and cSNPs documented in the blood droup antigen gene mutation database. *Human Mutation*.

[19] Haldane JBS (1949). Disease and evolution. Symposium sui fattori ecologici e genetici della speciazione negli animali. *Selected Genetic Papers of J. B. S. Haldane*.

[20] Hirschfeld L, Hirschfeld H (1919). Serological differences between the blood of different races. The result of researches on the macedonian front. *The Lancet*.

[21] Saitou N, Yamamoto FI (1997). Evolution of primate ABO blood group genes and their homologous genes. *Molecular Biology and Evolution*.

[22] Calafell F, Roubinet F, Ramírez-Soriano A, Saitou N, Bertranpetit J, Blancher A (2008). Evolutionary dynamics of the human ABO gene. *Human Genetics*.

[23] Segurel L, Thompson EE, Flutre T (2012). The ABO blood group is a trans-species polymorphism in primates. *Proceedings of the National Academy of Sciences of the United States of America*.

[24] Ferrer-Admetlla A, Sikora M, Laayouni H (2009). A natural history of FUT2 polymorphism in humans. *Molecular Biology and Evolution*.

[25] Fry AE, Griffiths MJ, Auburn S (2008). Common variation in the ABO glycosyltransferase is associated with susceptibility to severe Plasmodium falciparum malaria. *Human Molecular Genetics*.

[26] Rowe JA, Handel IG, Thera MA (2007). Blood group O protects against severe Plasmodium falciparum malaria through the mechanism of reduced rosetting. *Proceedings of the National Academy of Sciences of the United States of America*.

[27] Lindesmith L, Moe C, Marionneau S (2003). Human susceptibility and resistance to Norwalk virus infection. *Nature Medicine*.

[28] Ruiz-Palacios GM, Cervantes LE, Ramos P, Chavez-Munguia B, Newburg DS (2003). Campylobacter jejuni binds intestinal H(O) antigen (Fuc*α*1, 2Gal*β*1, 4GlcNAc), and fucosyloligosaccharides of human milk inhibit its binding and infection. *Journal of Biological Chemistry*.

[29] Boren T, Falk P, Roth KA, Larson G, Normark S (1993). Attachment of Helicobacter pylori to human gastric epithelium mediated by blood group antigens. *Science*.

[30] Harris JB, Khan AI, LaRocque RC (2005). Blood group, immunity, and risk of infection with Vibrio cholerae in an area of endemicity. *Infection and Immunity*.

[31] Aspholm-Hurtig M, Dailide G, Lahmann M (2004). Functional adaptation of BabA the *H. pylori* ABO blood group antigen binding adhesin. *Science*.

[32] Imberty A, Varrot A (2008). Microbial recognition of human cell surface glycoconjugates. *Current Opinion in Structural Biology*.

[33] Fumagalli M, Pozzoli U, Cagliani R (2010). Genome-wide identification of susceptibility alleles for viral infections through a population genetics approach. *PLoS Genetics*.

[34] Rojek JM, Spiropoulou CF, Campbell KP, Kunz S (2007). Old world and clade C new world arenaviruses mimic the molecular mechanism of receptor recognition used by *α*-dystroglycan’s host-derived ligands. *Journal of Virology*.

[35] Kunz S, Rojek JM, Kanagawa M (2005). Posttranslational modification of *α*-dystroglycan, the cellular receptor for arenaviruses, by the glycosyltransferase LARGE is critical for virus binding. *Journal of Virology*.

[36] Sabeti PC, Varilly P, Fry B (2007). Genome-wide detection and characterization of positive selection in human populations. *Nature*.

[37] Andersen KG, Shylakhter I, Tabrizi S, Grossman SR, Happi CT, Sabeti PC (2012). Genome-wide scans provide evidence for positive selection of genes implicated in Lassa fever. *Philosophical Transactions of the Royal Society*.

[38] Yawata M, Yawata N, Draghi M, Little AM, Partheniou F, Parham P (2006). Roles for HLA and KIR polymorphisms in natural killer cell repertoire selection and modulation of effector function. *Journal of Experimental Medicine*.

[39] Uhrberg M, Valiante NM, Shum BP (1997). Human diversity in killer cell inhibitory receptor genes. *Immunity*.

[40] Hughes AL, Nei M (1989). Nucleotide substitution at major histocompatibility complex class II loci: evidence for overdominant selection. *Proceedings of the National Academy of Sciences of the United States of America*.

[41] Hughes AL, Nei M (1988). Pattern of nucleotide substitution at major histocompatibility complex class I loci reveals overdominant selection. *Nature*.

[42] Dean M, Carrington M, O’Brien SJ (2002). Balanced polymorphism selected by genetic versus infectious human disease. *Annual Review of Genomics and Human Genetics*.

[43] Takahata N, Nei M (1990). Allelic genealogy under overdominant and frequency-dependent selection and polymorphism of major histocompatibility complex loci. *Genetics*.

[44] Satta Y, O’Huigin C, Takahata N, Klein J (1994). Intensity of natural selection at the major histocompatibility complex loci. *Proceedings of the National Academy of Sciences of the United States of America*.

[45] Takahata N, Satta Y, Klein J (1992). Polymorphism and balancing selection at major histocompatibility complex loci. *Genetics*.

[46] Miretti MM, Walsh EC, Ke X (2005). A high-resolution linkage-disequilibrium map of the human major histocompatibility complex and first generation of tag single-nucleotide polymorphisms. *American Journal of Human Genetics*.

[47] Mayer WE, Jonker M, Klein D, Ivanyi P, van Seventer G, Klein J (1988). Nucleotide sequences of chimpanzee MHC class I alleles: evidence for trans-species mode of evolution. *The EMBO Journal*.

[48] Ayala FJ (1995). The myth of Eve: molecular biology and human origins. *Science*.

[49] Prugnolle F, Manica A, Charpentier M, Guégan JF, Guernier V, Balloux F (2005). Pathogen-driven selection and worldwide HLA class I diversity. *Current Biology*.

[50] Chaix R, Cao C, Donnelly P (2008). Is mate choice in humans MHC-dependent?. *PLoS Genetics*.

[51] Derti A, Cenik C, Kraft P, Roth FP (2010). Absence of evidence for MHC-dependent mate selection within HapMap populations. *PLoS Genetics*.

[52] Saveanu L, Carroll O, Lindo V (2005). Concerted peptide trimming by human ERAP1 and ERAP2 aminopeptidase complexes in the endoplasmic reticulum. *Nature Immunology*.

[53] Andrés AM, Dennis MY, Kretzschmar WW (2010). Balancing selection maintains a form of ERAP2 that undergoes nonsense-mediated decay and affects antigen presentation. *PLoS Genetics*.

[54] Cagliani R, Riva S, Biasin M (2010). Genetic diversity at endoplasmic reticulum aminopeptidases is maintained by balancing selection and is associated with natural resistance to HIV-1 infection. *Human Molecular Genetics*.

[55] Kim S, Lee S, Shin J (2011). Human cytomegalovirus microRNA miR-US4-1 inhibits CD8(^+^) T cell responses by targeting the aminopeptidase ERAP1. *Nature Immunology*.

[56] Brass AL, Huang IC, Benita Y (2009). The IFITM proteins mediate cellular resistance to influenza A H1N1 virus, West Nile Virus, and Dengue Virus. *Cell*.

[57] Everitt AR, Clare S, Pertel T (2012). IFITM3 restricts the morbidity and mortality associated with influenza. *Nature*.

[58] Sabeti PC, Schaffner SF, Fry B (2006). Positive natural selection in the human lineage. *Science*.

[59] Voight BF, Kudaravalli S, Wen X, Pritchard JK (2006). A map of recent positive selection in the human genome. *PLoS Biology*.

[60] Williamson SH, Hubisz MJ, Clark AG, Payseur BA, Bustamante CD, Nielsen R (2007). Localizing recent adaptive evolution in the human genome. *PLoS Genetics*.

[61] Barreiro LB, Laval G, Quach H, Patin E, Quintana-Murci L (2008). Natural selection has driven population differentiation in modern humans. *Nature Genetics*.

[62] Tang K, Thornton KR, Stoneking M (2007). A new approach for using genome scans to detect recent positive selection in the human genome. *PLoS Biology*.

[63] Andrés AM, Hubisz MJ, Indap A (2009). Targets of balancing selection in the human genome. *Molecular Biology and Evolution*.

[64] Vasseur E, Boniotto M, Patin E (2012). The evolutionary landscape of cytosolic microbial sensors in humans. *The American Journal of Human Genetics*.

[65] Witola WH, Mui E, Hargrave A (2011). NALP1 influences susceptibility to human congenital toxoplasmosis, proinflammatory cytokine response, and fate of Toxoplasma gondii-infected monocytic cells. *Infection and Immunity*.

[66] Swanberg M, Lidman O, Padyukov L (2005). MHC2TA is associated with differential MHC molecule expression and susceptibility to rheumatoid arthritis, multiple sclerosis and myocardial infarction. *Nature Genetics*.

[67] Jostins L, Ripke S, Weersma RK (2012). Host-microbe interactions have shaped the genetic architecture of inflammatory bowel disease. *Nature*.

